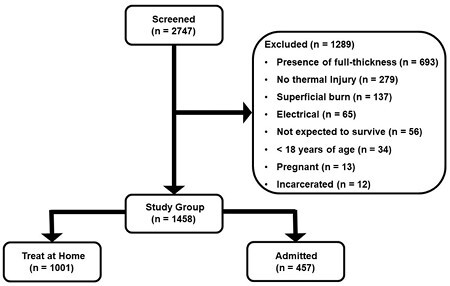# 121 A Retrospective, Non-inferiority Treat-at-Home Study Utilizing a Surfactant-based Dressing for Partial-thickness Burn Wounds (HOME)

**DOI:** 10.1093/jbcr/irae036.120

**Published:** 2024-04-17

**Authors:** Elysha Lyle, Jacob T Jarreau, Matthew Sanders, Maria Tucci Jones, Denise Knight, Patrick Brockway, Mahmoud Hassouba, Sai R Velamuri, David M Hill

**Affiliations:** Regional One Health Firefighter's Burn Center, Memphis, Tennessee; Regional One Health, Memphis, Tennessee; Regional One Health Firefighters Burn Center, Collierville, Tennessee; ROH Firefighters Burn Center, New Albany, Mississippi; Firefighters Burn Center, Regional One Health, Memphis, TN; Regional One Health Firefighter's Burn Center, Memphis, Tennessee; Regional One Health, Memphis, Tennessee; Regional One Health Firefighters Burn Center, Collierville, Tennessee; ROH Firefighters Burn Center, New Albany, Mississippi; Firefighters Burn Center, Regional One Health, Memphis, TN; Regional One Health Firefighter's Burn Center, Memphis, Tennessee; Regional One Health, Memphis, Tennessee; Regional One Health Firefighters Burn Center, Collierville, Tennessee; ROH Firefighters Burn Center, New Albany, Mississippi; Firefighters Burn Center, Regional One Health, Memphis, TN; Regional One Health Firefighter's Burn Center, Memphis, Tennessee; Regional One Health, Memphis, Tennessee; Regional One Health Firefighters Burn Center, Collierville, Tennessee; ROH Firefighters Burn Center, New Albany, Mississippi; Firefighters Burn Center, Regional One Health, Memphis, TN; Regional One Health Firefighter's Burn Center, Memphis, Tennessee; Regional One Health, Memphis, Tennessee; Regional One Health Firefighters Burn Center, Collierville, Tennessee; ROH Firefighters Burn Center, New Albany, Mississippi; Firefighters Burn Center, Regional One Health, Memphis, TN; Regional One Health Firefighter's Burn Center, Memphis, Tennessee; Regional One Health, Memphis, Tennessee; Regional One Health Firefighters Burn Center, Collierville, Tennessee; ROH Firefighters Burn Center, New Albany, Mississippi; Firefighters Burn Center, Regional One Health, Memphis, TN; Regional One Health Firefighter's Burn Center, Memphis, Tennessee; Regional One Health, Memphis, Tennessee; Regional One Health Firefighters Burn Center, Collierville, Tennessee; ROH Firefighters Burn Center, New Albany, Mississippi; Firefighters Burn Center, Regional One Health, Memphis, TN; Regional One Health Firefighter's Burn Center, Memphis, Tennessee; Regional One Health, Memphis, Tennessee; Regional One Health Firefighters Burn Center, Collierville, Tennessee; ROH Firefighters Burn Center, New Albany, Mississippi; Firefighters Burn Center, Regional One Health, Memphis, TN; Regional One Health Firefighter's Burn Center, Memphis, Tennessee; Regional One Health, Memphis, Tennessee; Regional One Health Firefighters Burn Center, Collierville, Tennessee; ROH Firefighters Burn Center, New Albany, Mississippi; Firefighters Burn Center, Regional One Health, Memphis, TN

## Abstract

**Introduction:**

Partial thickness (PT) burn injuries are the most common depth of burn seen in the emergency department. While some deep partial thickness injuries will eventually require surgery, the many PT wounds can be successfully managed as an outpatient, given immediate care, education, and supplies. The primary hypothesis was a treat-at-home (TH) strategy, particularly with a surfactant-based dressing (WSD), would result in no greater risk of requiring surgical intervention for PT wounds. Second, we hypothesized there to be a bias toward surgery in the admitted patients. Lastly, a TH strategy results in significant cost savings.

**Methods:**

This single-center, retrospective study was dual IRB approved and included all patients treated in the burn center emergency department (ED) between May 2019 and May 2023. Patients were excluded for having superficial burns, full-thickness (FT) burns, electrical burn, lack of burn, less than 18 years old, pregnant or incarcerated, or not expected to survive. The years were chosen based on sample size analysis and estimated ED visits. The planned enrollment was designed to fulfill non-inferiority and potential superiority of surfactant-based dressing versus any another TH strategy. Chi-square test was used to determine a difference in number admitted for subsequent surgery. Sigmaplot 15.0 was used for statistical analysis.

**Results:**

Two-thousand forty-seven patients were screened. After exclusions, 1,458 patients were included in the study group (Figure 1) with two-thirds in the TH group. About half were sent home with WSD vs another dressing (CTL). There were no differences in age, sex, race, medical history, or mechanism of burn. The CTL patients were more likely to be cocaine positive and have a burn involving the head / neck. WSD had larger burns and more likely to have deep PT and extremity burns, and a longer delay from injury. Few in WSD and CTL required subsequent admission for surgery [5 (1.0%) vs 2 (0.4%)]. Although, CTL had significantly more not return for follow up [153 (31.3%) vs 239 (46.7%)]. Patients admitted from the ED were more likely to be older, male, Caucasian, complex medical and social history, inhalation injury, presence of deep PT and larger burns, transfer from another facility, and present with infection. For those admitted, only 36.8% received surgery with a 3 day median time to first operation. The median length of stay for patients not requiring surgery was 1 day. Assuming the presence of social barriers in 1/3 and only considering bed cost, allowing capable patients with PT to treat-at-home would save an estimated annual $3.4 million.

**Conclusions:**

This is the largest study to evaluate the use of WSD for immediate treatment of PT burns to avoid admission. No matter the choice of home wound treatment, few required subsequent admission for surgery. A TH model could result in millions of dollars of savings.

**Applicability of Research to Practice:**

A treat-at-home model could save the millions of dollars for patients and hospitals